# Supplementary material for: First-year dynamics of the anaerobic microbiome and archaeome in infants’ oral and gastrointestinal systems
Source: mSystems. 2024 Dec 23;10(1):e01071-24. doi: 10.1128/msystems.01071-24 (PMC11756582; doi:10.1128/msystems.01071-24)
Supplement: Supplemental Figures — Figures S1 to S21. [file msystems.01071-24-s0001.pdf]

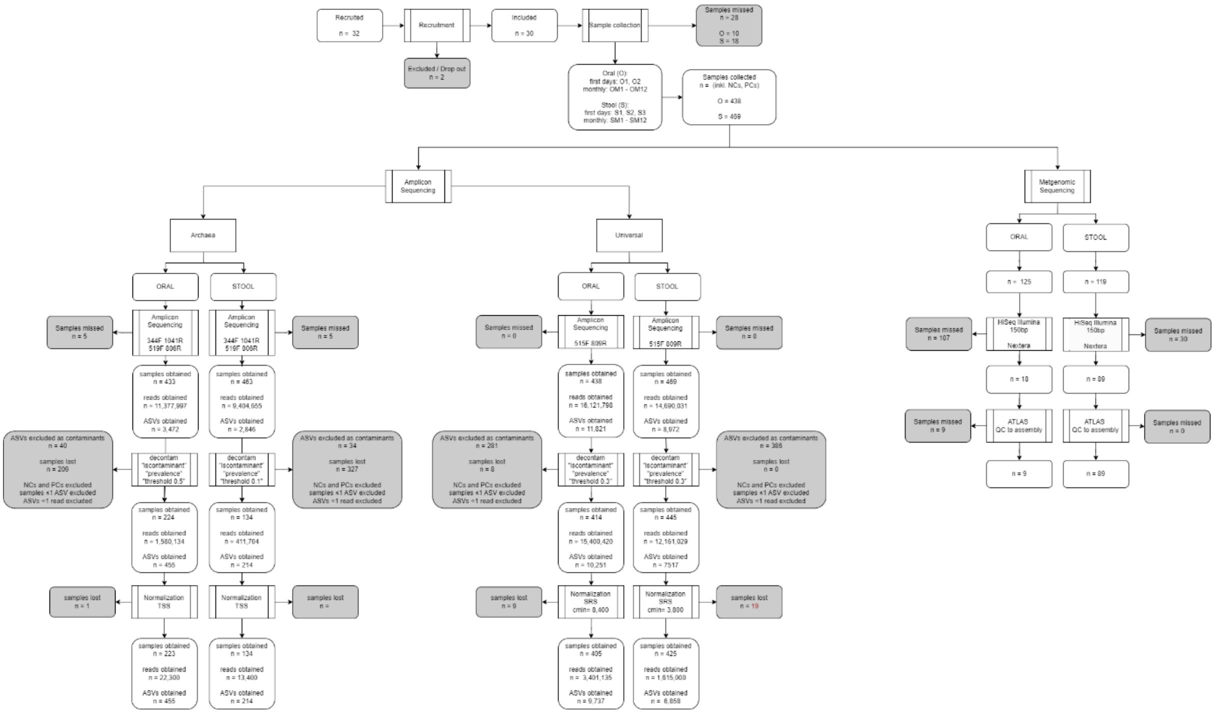

Suppl. Fig. 1: A detailed overview of the number of samples, reads and ASVs obtained from recruiting over sample processing to data obtained.

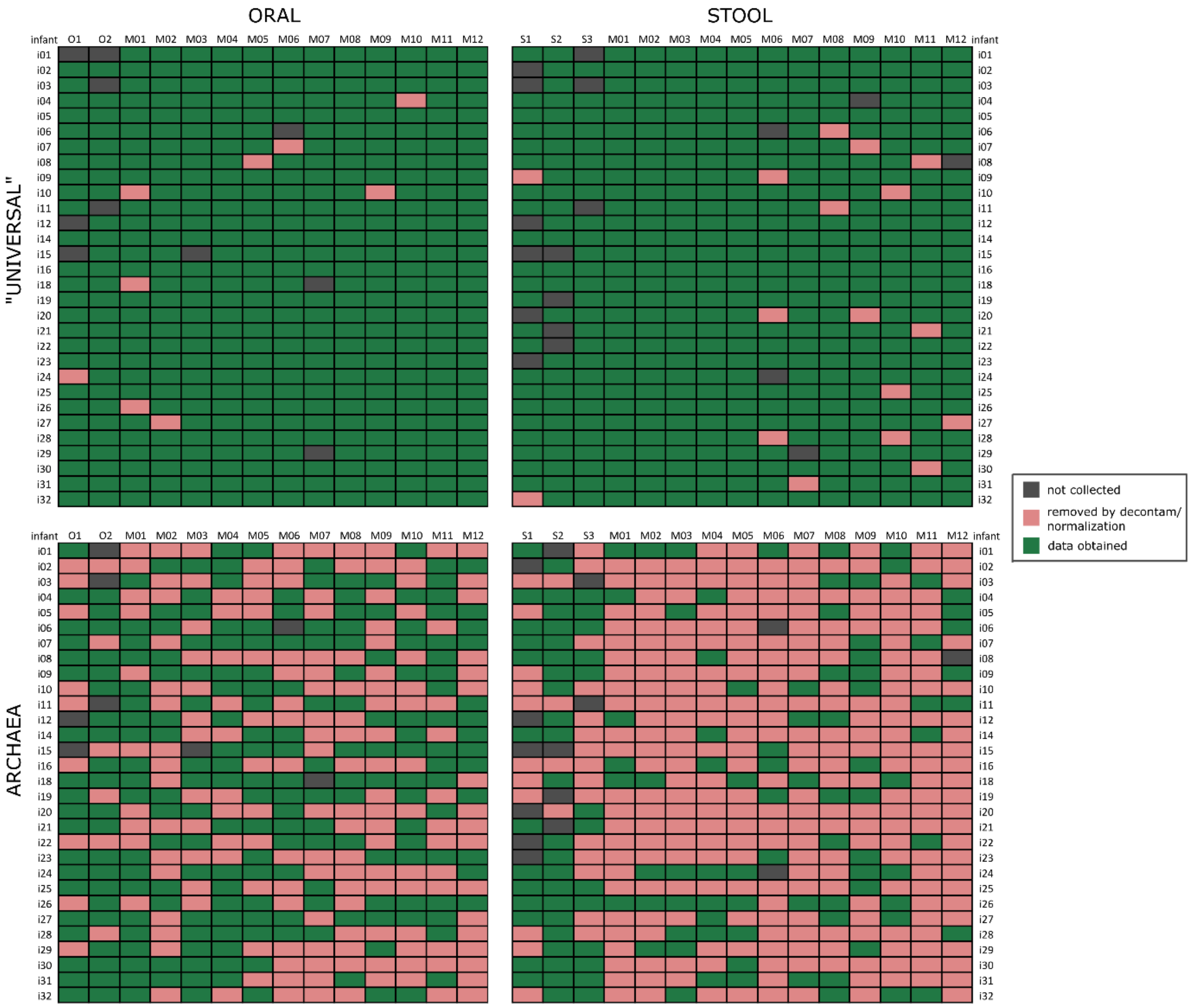

Suppl. Fig. 2: An overview of data obtained for “universal” and archaeal amplicon sequencing approach of oral and stool samples. For each infant (rows) and time point (columns) it is indicated if samples could not be collected (gray), were lost during data processing (pink) or could successfully be obtained (green)

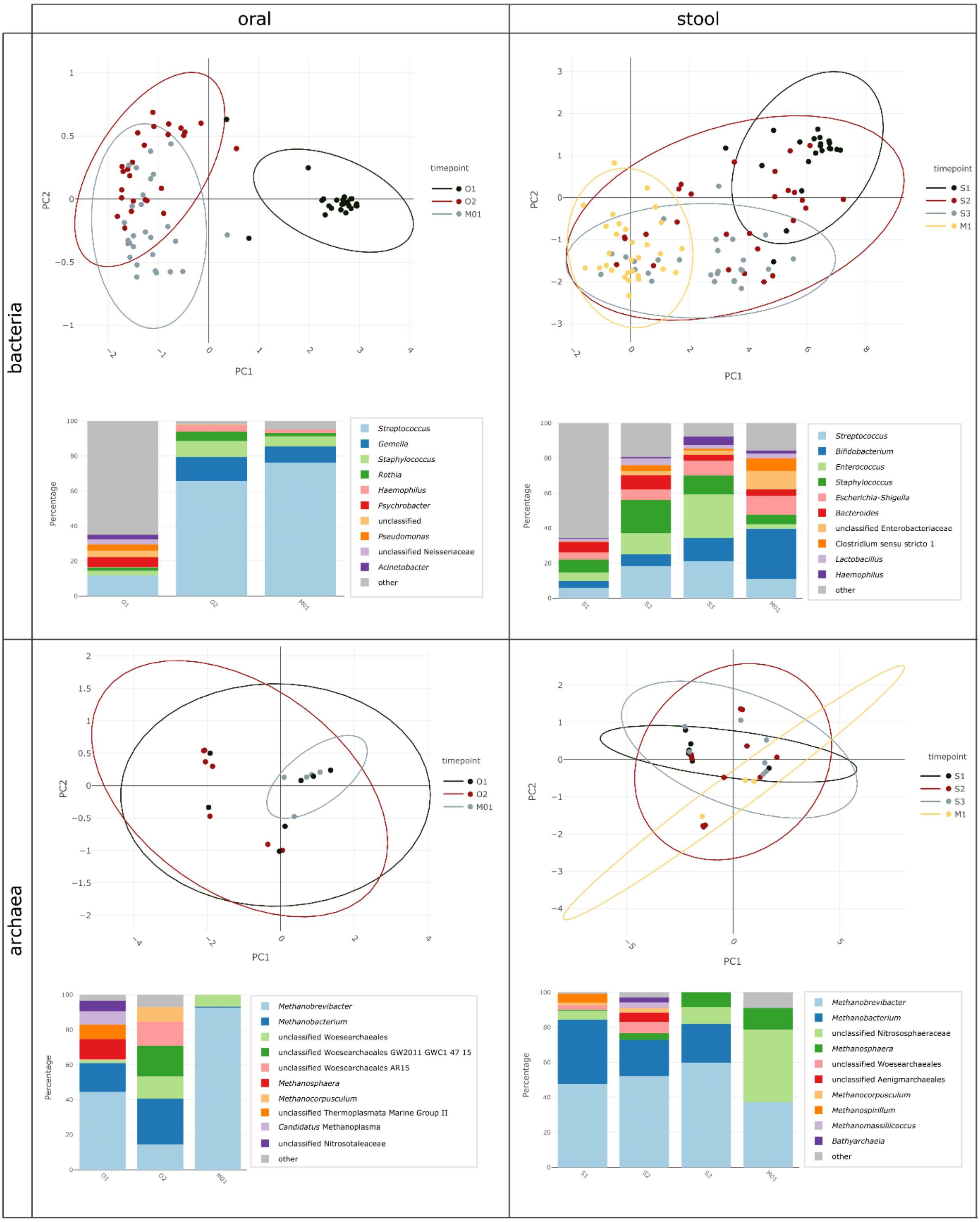

Suppl. Fig. 3: PC plots and stacked bar plots of early life samples of stool (S1, S2, S3) and oral samples (=1, O2) in comparison to the respective samples taken at month M01; data are separated by “universal” and archaeal amplicon sequencing approach and sample type (stool and oral); in each stacked bar plot the top 10 abundant archaeal or bacterial genera are presented.

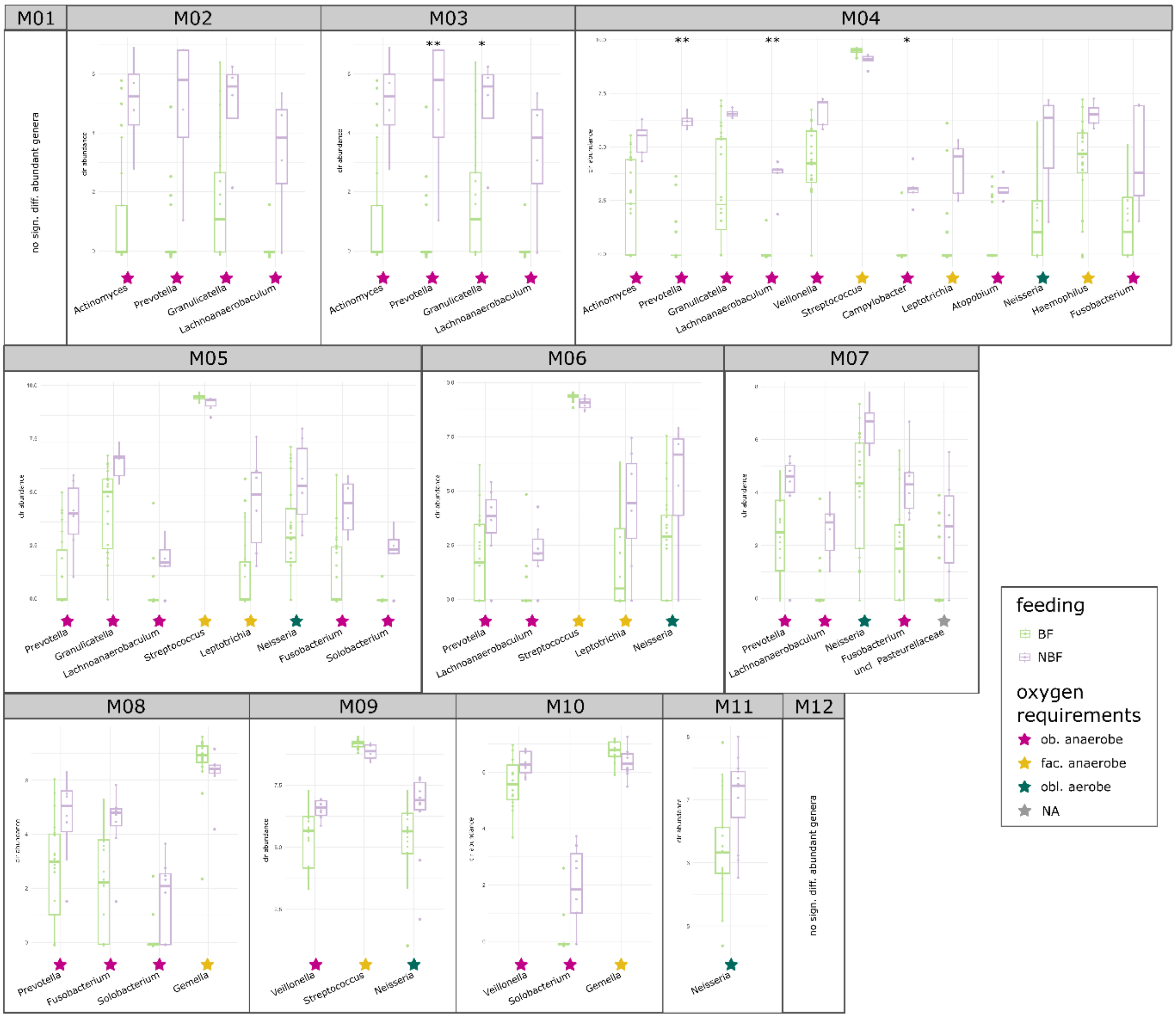

Suppl. Fig. 4: Differentially abundant bacterial genera in the oral microbiome per time point (months M01 to M12) between breastfed (BF = light green) and non-breastfed (NBF = lavender) infants, performed by Aldex2. Selection was based on the significant p-value of Aldex2 ($p < 0.05$); significance asterisks indicate BH (Benjamini Hochberg) corrected *p*-values ($q < 0.05$). The oxygen requirement of the respective genera is highlighted by colored stars: pink: obligate anaerobes, yellow: facultative anaerobes, petrol: obligate aerobes.

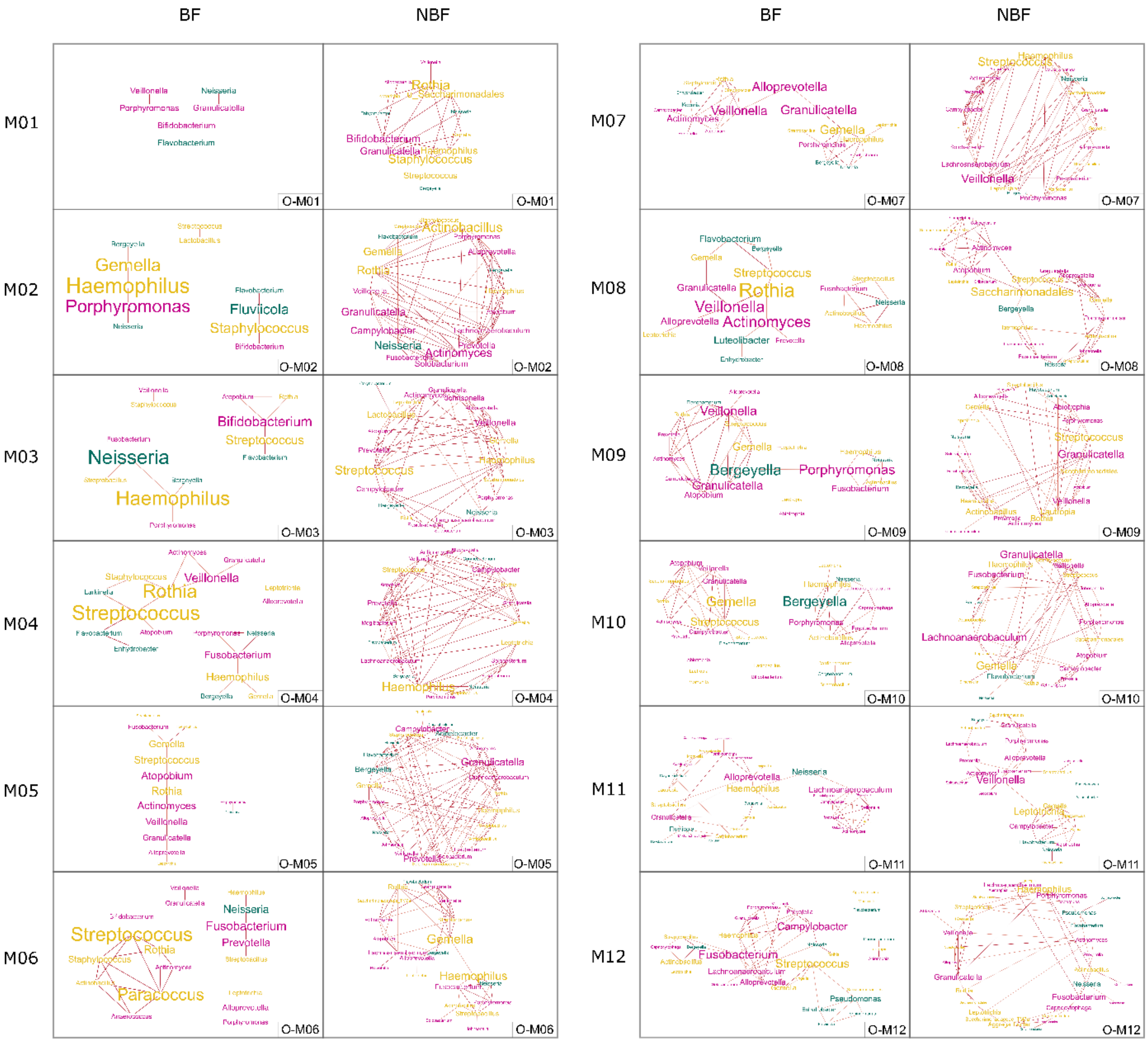

Suppl. Fig. 5: Network on oral samples of BF and NBF infants. Per time point (months M01 to M12), one network is depicted. Font size indicates stress centrality, colors indicate oxygen requirement: pink: obligate anaerobes, yellow: facultative anaerobe, petrol: obligate aerobes.

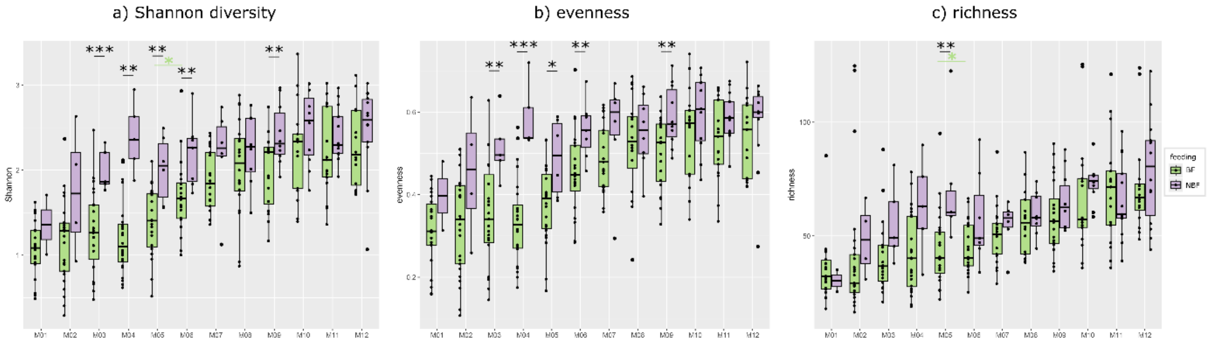

Suppl. Fig. 6: Alpha diversity of oral samples depicted for breastfed (BF =light green) and non-breastfed (NBF = lavender) infants with asterisks indicating significant difference (*q* - values) between those two groups. a) Shannon diversity, b) evenness, c) richness. Significant *q* - values between tps in BF infants are indicated with light green asterisks.

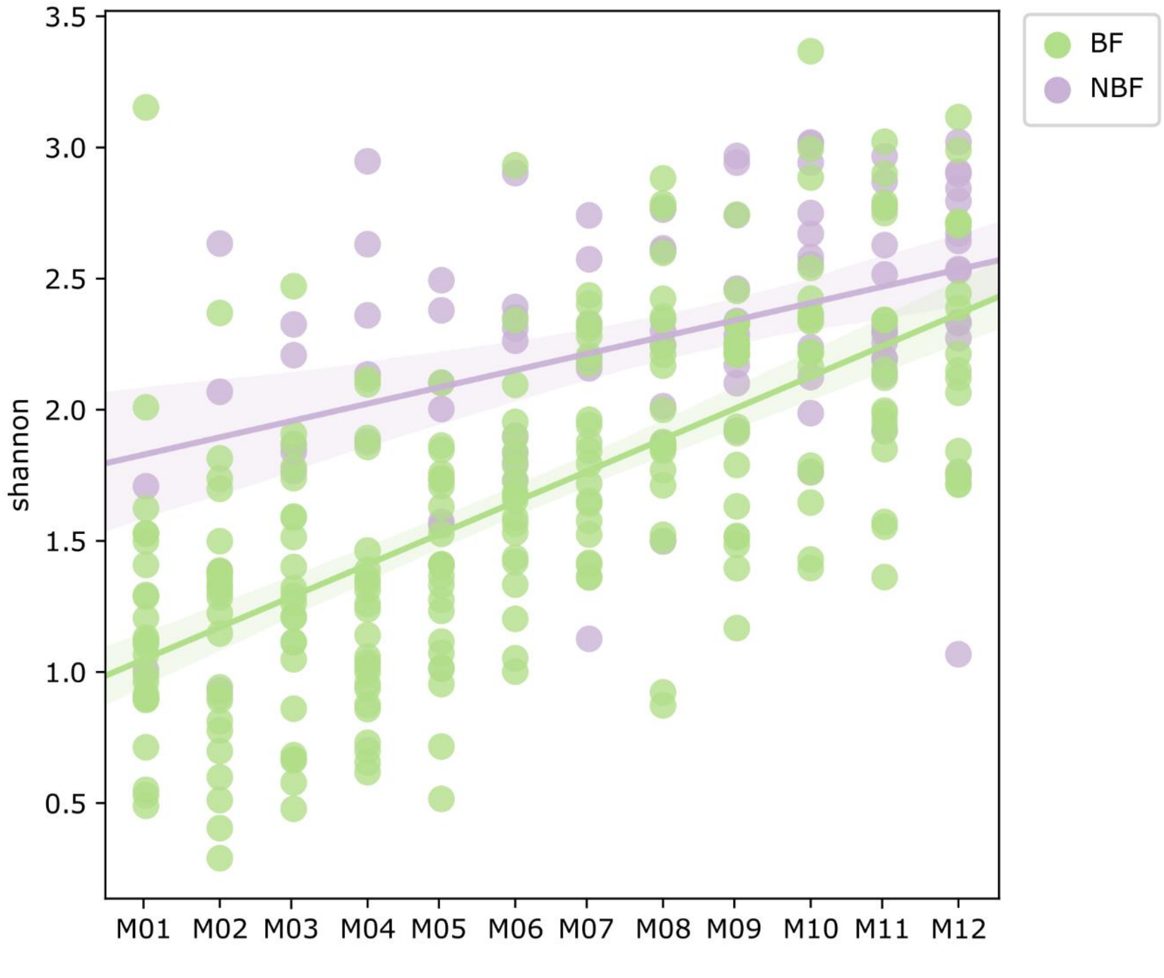

Suppl. Fig. 7: Longitudinal linear mixed effect (LME) for Shannon diversity of oral samples

per time point (months M01 to M12); data are separated for breastfed (BF = light green) and non-breastfed (NBF) infants.

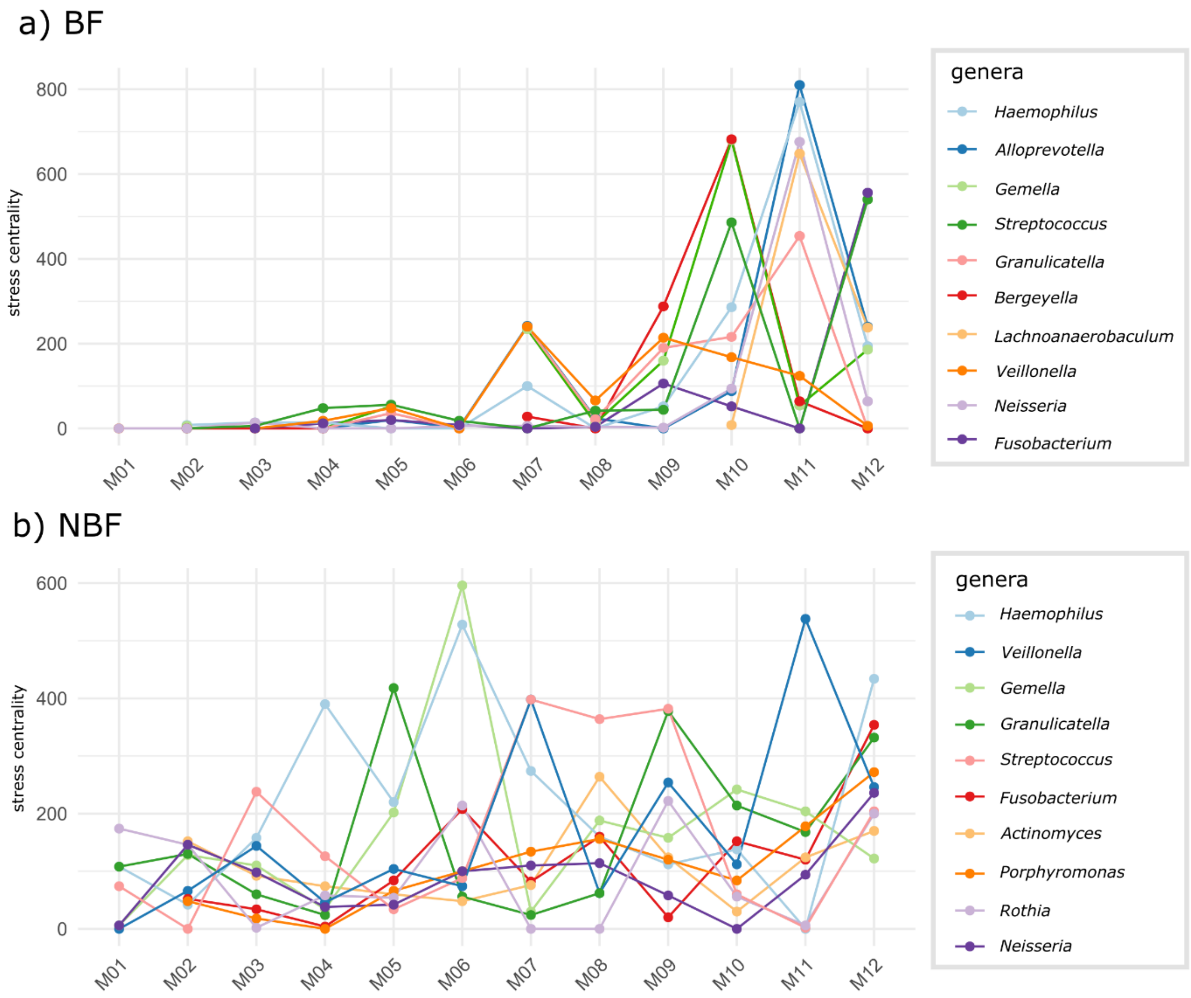

Suppl. Fig. 8: Stress centrality in the oral microbiome networks of top 10 bacterial genera per time point (months M01 to M12); data are shown separately for a) breastfed (BF) and b) non-breastfed (NBF) infants.

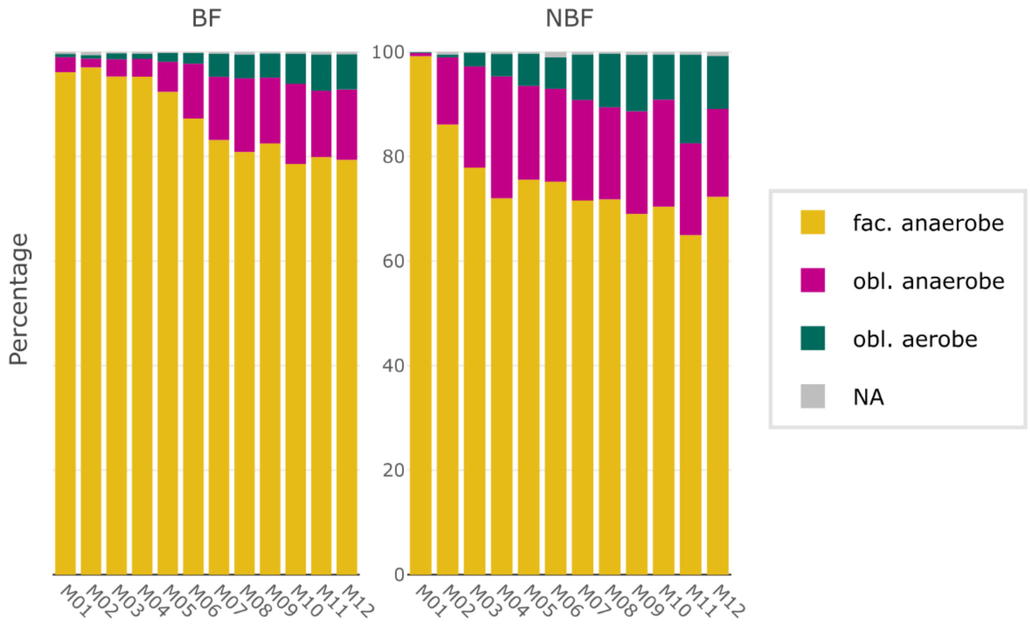

Suppl. Fig. 9: Stacked bar plots of relative abundances of oral bacterial taxa per time point (months M01 to M12), colored by their oxygen requirements: pink: obligate anaerobes, yellow: facultative anaerobes, petrol: obligate aerobes, grey: not assigned; data are separated for breastfed (BF) and non-breastfed (NBF) infants.

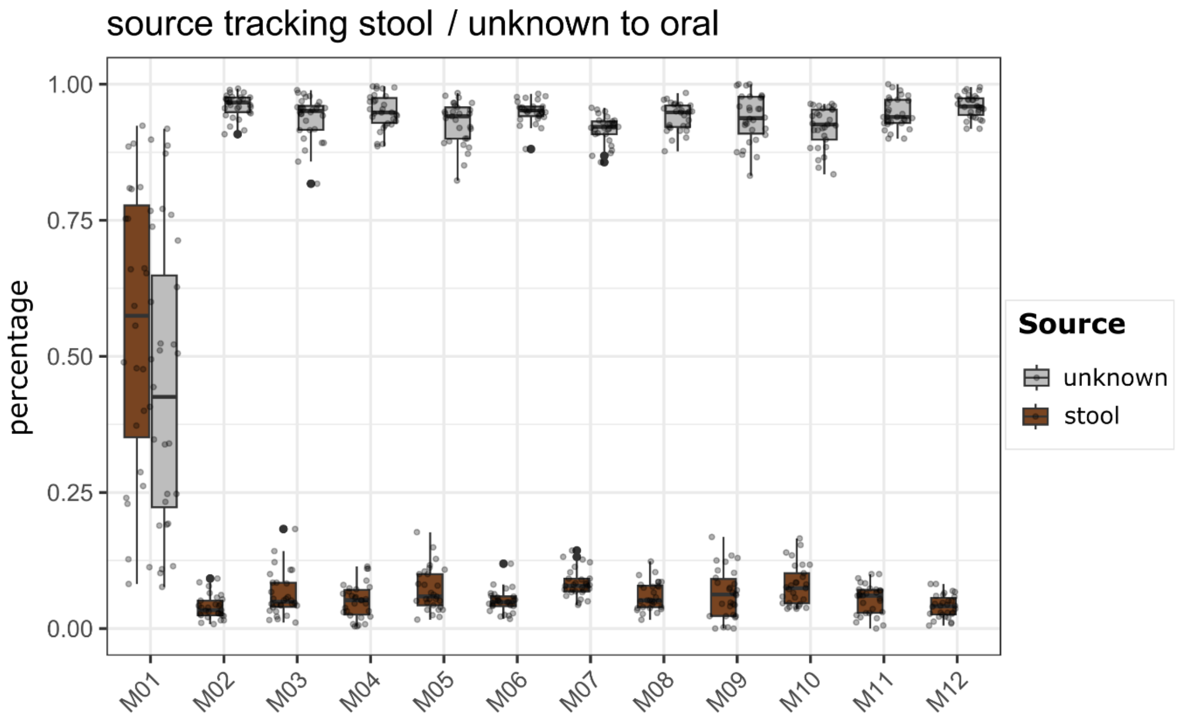

Suppl. Fig. 10: Source Tracking probability of bacterial taxa being transferred from gut and unknown sources to oral as a sink, depicted per time point (months M01 to M12).

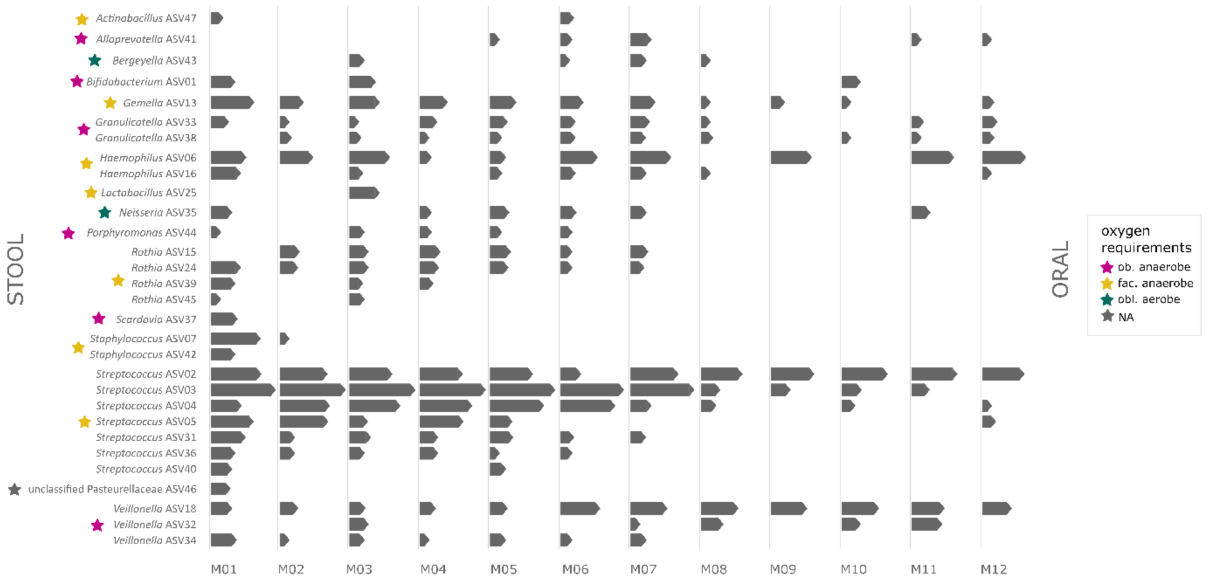

Suppl. Fig. 11: Source Tracking of specific bacterial taxa being transferred from gut source to oral sink. The top30 ASVs are depicted per time point (months M01 to M12), the length of the bars indicates the log-transformed counts of a taxa. The oxygen requirement of the

respective ASV is highlighted by colored stars: pink: obligate anaerobes, yellow: facultative anaerobes, petrol: obligate aerobes.

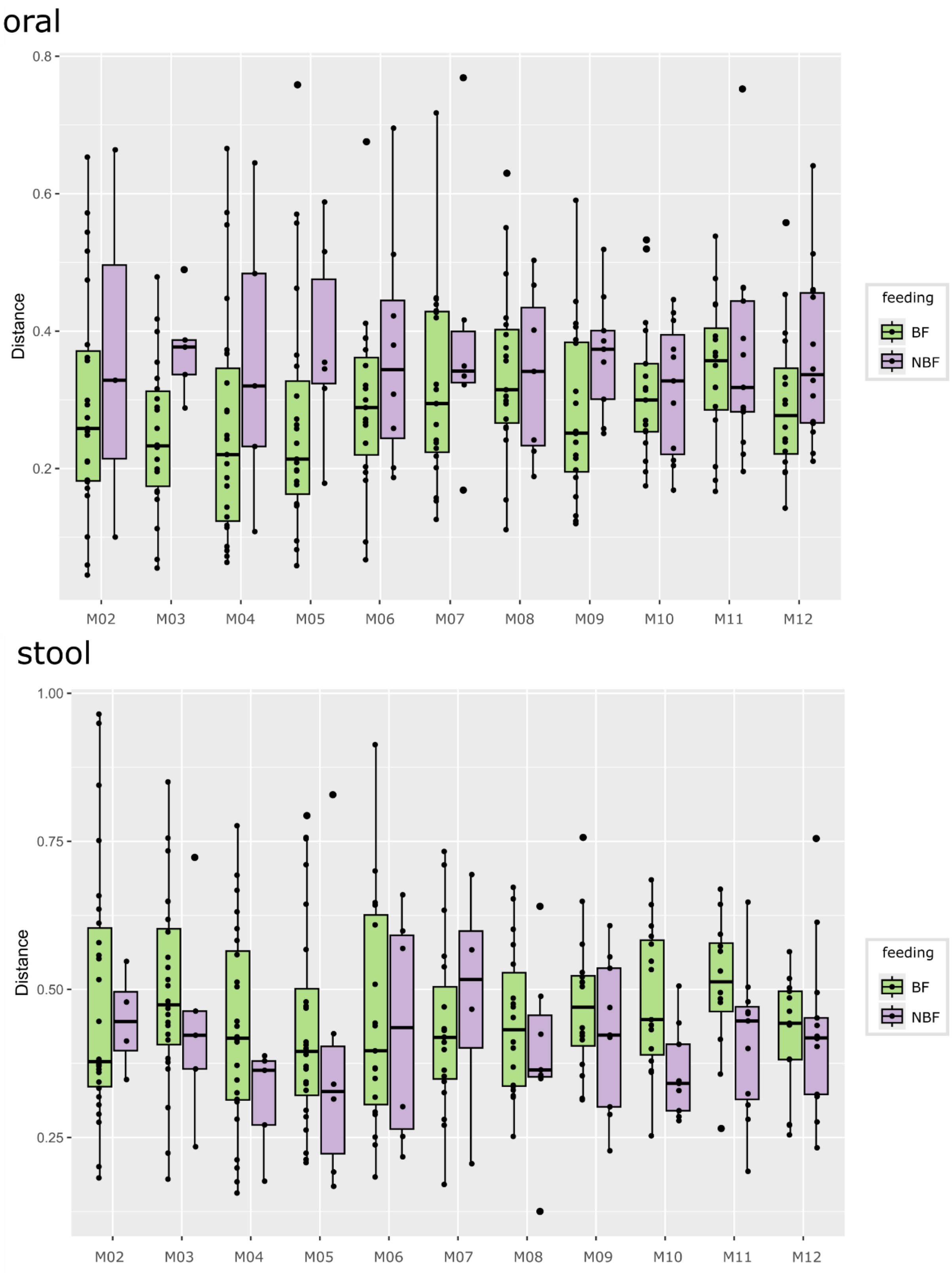

Suppl. Fig. 12: Longitudinal linear mixed effect (LME) on oral and stool data displaying Bray Curtis distance within one infant from one time point (tp) to the consecutive tp, with

M01 as reference; colors indicate breastfed (BF = light green) or non-breastfed (NBF = lavender) infants.

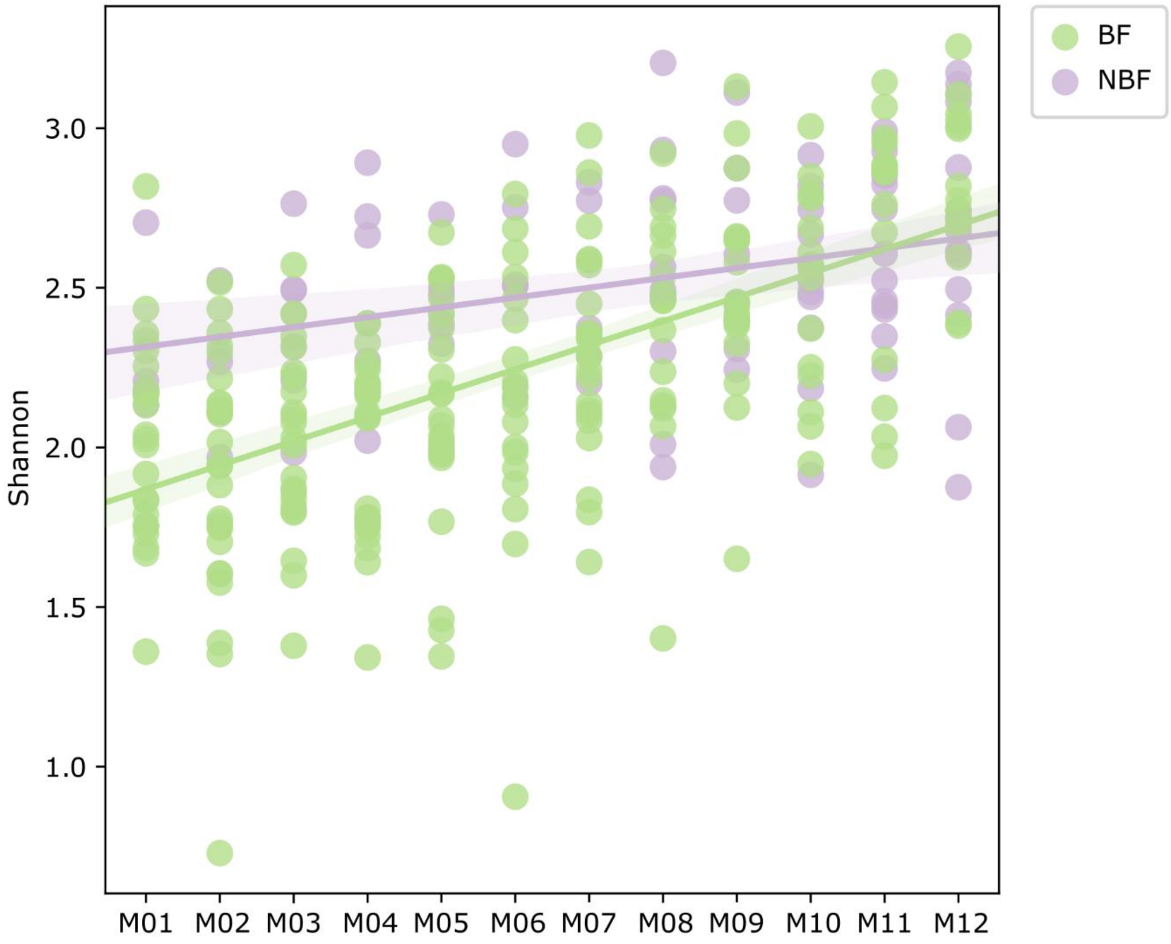

Suppl. Fig. 13: Longitudinal linear mixed effect (LME) for Shannon diversity of stool samples per time point (months M01 to M12); data are separated for breastfed (BF = light green) and non-breastfed (NBF) infants.

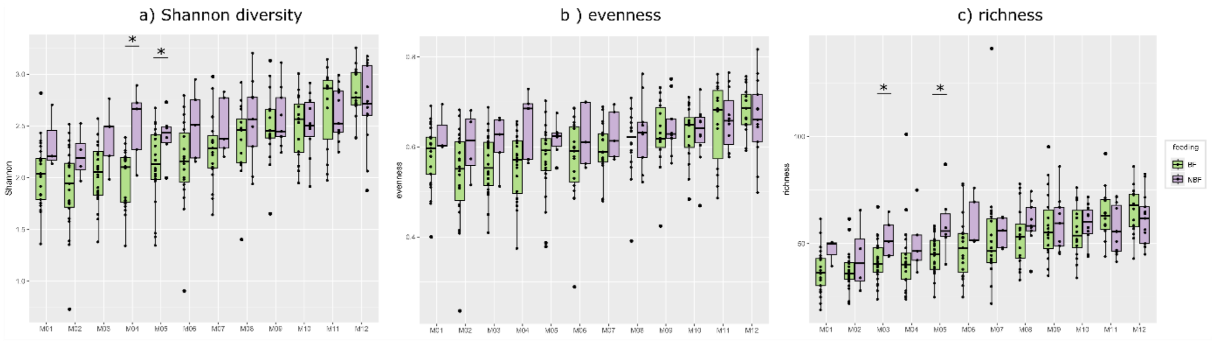

Suppl. Fig. 14: Alpha diversity of stool samples depicted for breastfed (BF =light green) and non-breastfed (NBF = lavender) infants with asterisks indicating significant difference (*q* - values) between those two groups. a) Shannon diversity, b) evenness, c) richness. No significant *q* - values between tps within one group (BF or NBF).

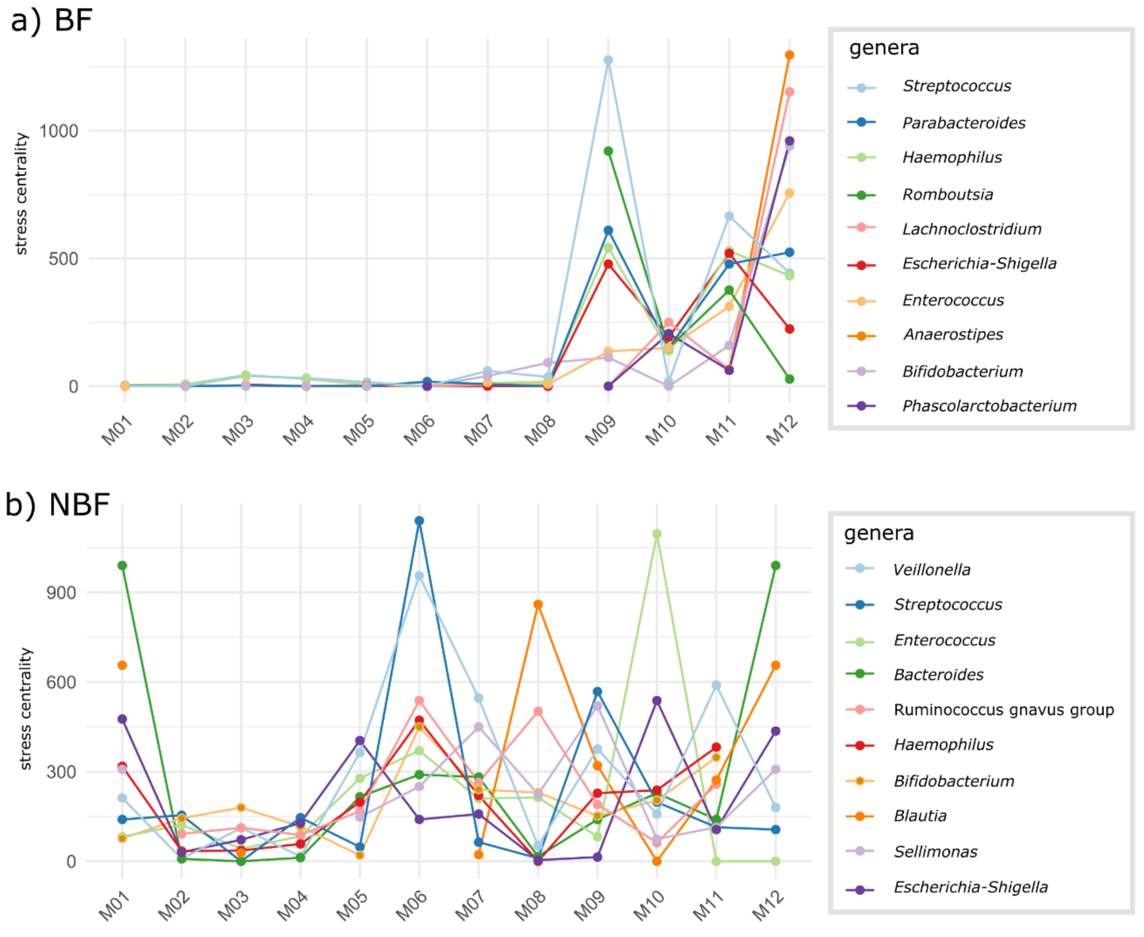

Suppl. Fig. 15: Stress centrality in the gut microbiome networks of top 10 bacterial genera

per time point (months M01 to M12); data are shown separately for a) breastfed (BF) and b) non-breastfed (NBF) infants.

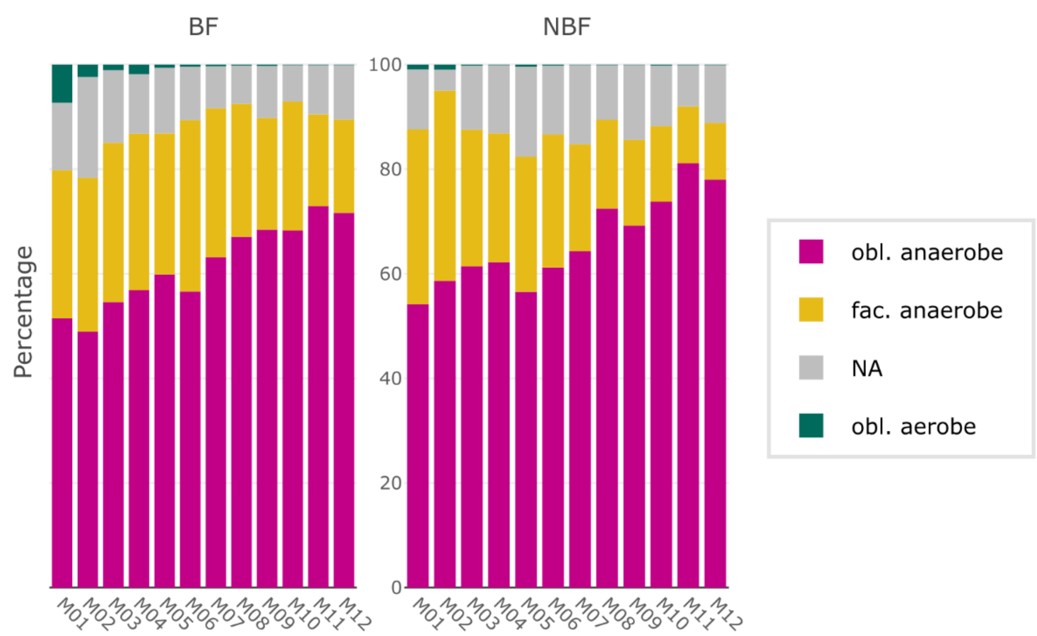

Suppl. Fig.

Suppl. Fig. 16: Stacked bar plots of relative abundances of bacterial taxa of the gut per time point (months M01 to M12), colored by their oxygen requirements: pink: obligate anaerobes, yellow: facultative anaerobes, petrol: obligate aerobes, gray: not assigned; data are separated for breastfed (BF) and non-breastfed (NBF) infants.

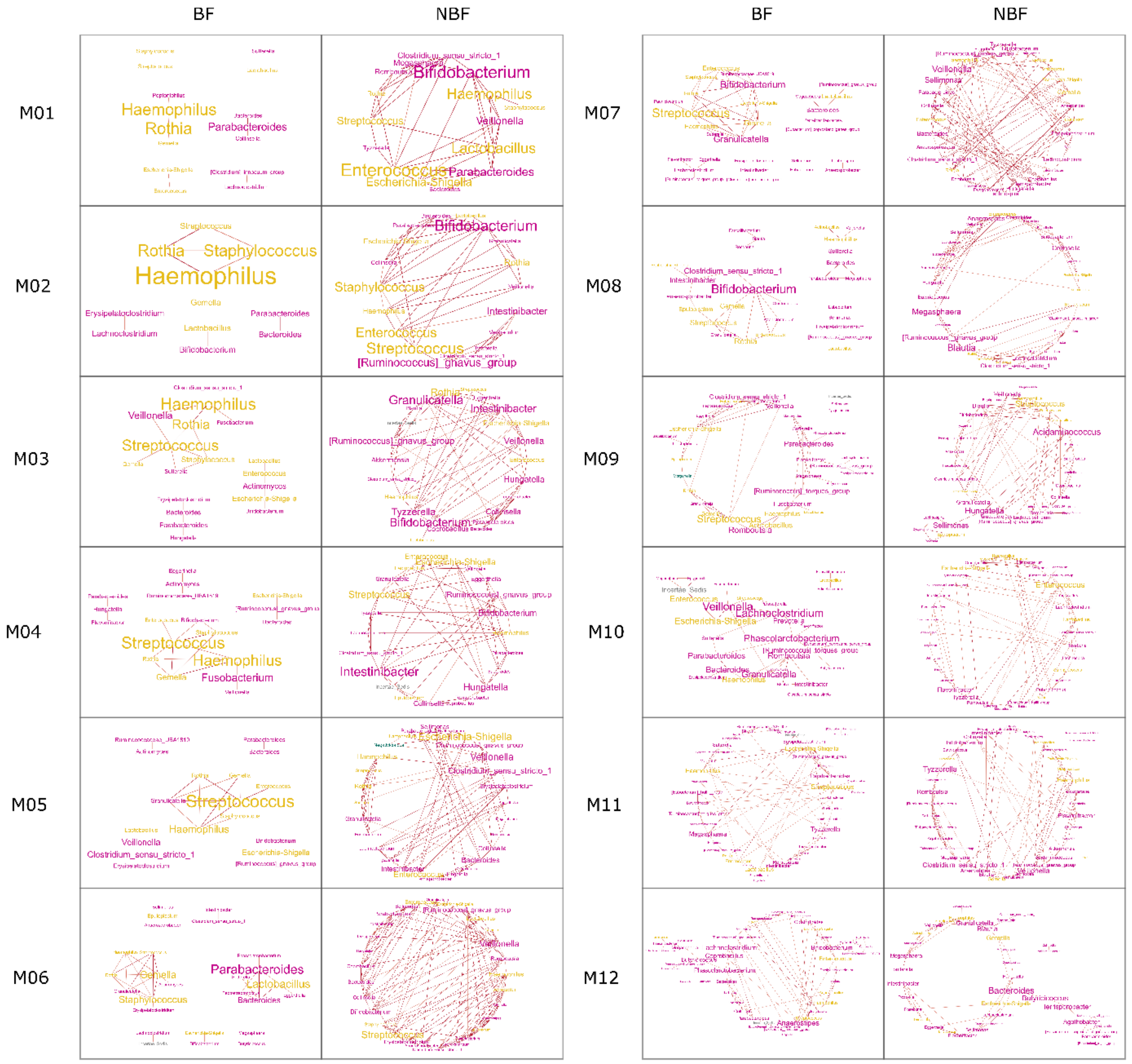

Suppl. Fig. 17: Network on stool samples of breastfed (BF) and non-breastfed (NBF) infants. Per time point, one network is depicted. Font size indicates stress centrality, colors indicate oxygen requirement: pink: obligate anaerobes, yellow: facultative anaerobe, petrol: obligate aerobes.

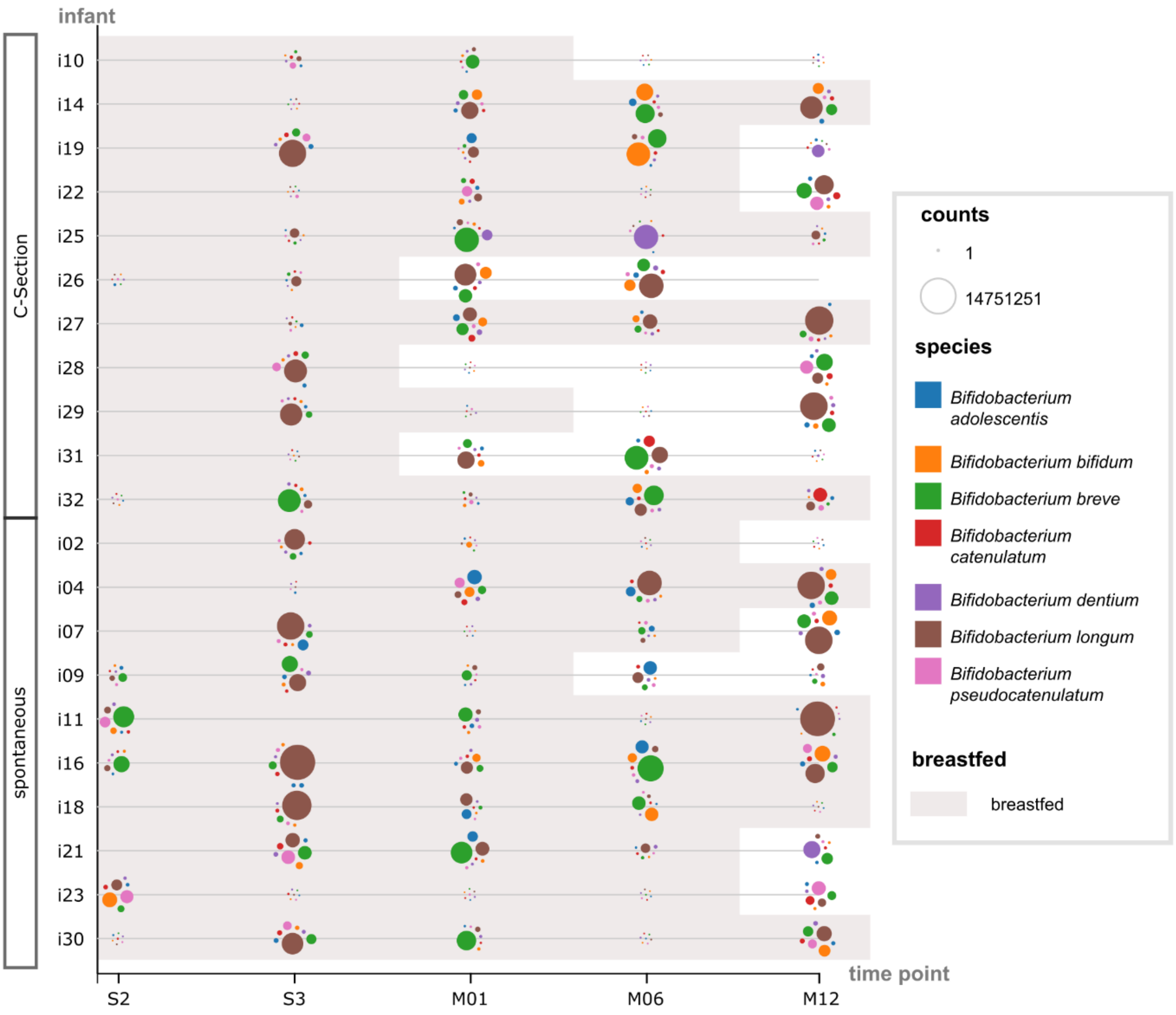

Suppl. Fig. 18: Beeswarm plot on Bifidobacterium species in stool samples for metagenomics sequencing for time points S2, S3 and months M01, M06 and M12; time points at which the infants were breastfed are underlaid with gray and infants are sorted by their mode of delivery.

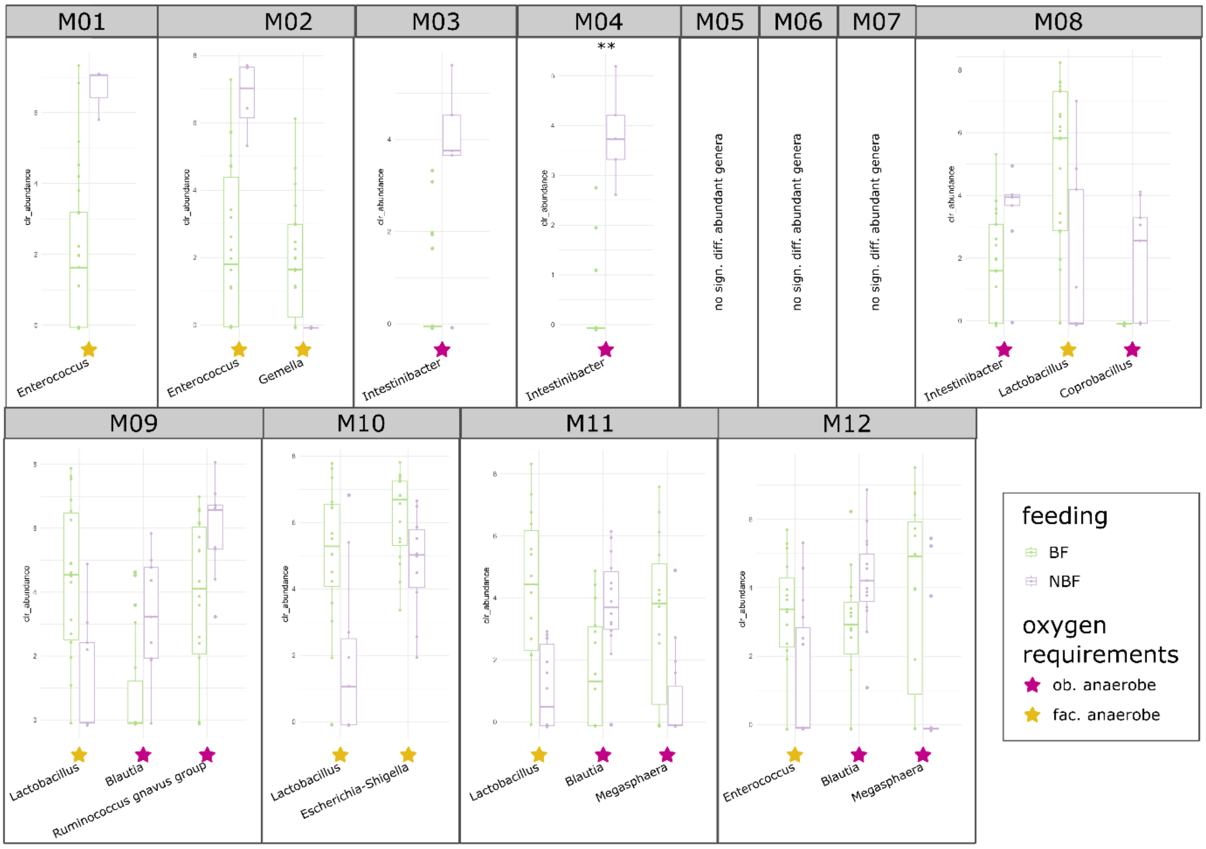

Suppl. Fig. 19: Differentially abundant bacterial genera in the gut microbiome per time point between breastfed (BF = light green) and non-breastfed (NBF = lavender) infants, performed by Aldex2. Selection was based on the significant *p*-values of Aldex2 ($p < 0.05$); significance asterisks indicate BH (Benjamini Hochberg) corrected *p*-values ($q < 0.05$). The oxygen requirement of the respective genera is highlighted by colored stars: pink: obligate anaerobes, yellow: facultative anaerobes, petrol: obligate aerobes.

a) M01

				KEGG	baseMean	log2FoldChange	sgnf
BF	Metabolism	Carbohydrate metabolism	Amino sugar & nucleotide sugar metabolism	K00992			*
			Starch and sucrose metabolism	K07405			*
		Glycan biosynthesis and metabolism	Glycosaminoglycan degradation	K10532			*
		Metabolism of other amino acids	D-Amino acid metabolism	K22210			**
	NA	Poorly characterized	Function unknown	K07063			***
				K09922			*
		Uncl.: metabolism	Enzymes with EC numbers	K18197			*
				K21575			*
		Uncl.: signaling and cellular processes	Structural proteins	K21571			*
	Protein families	Genetic information processing	Transcription factors	K19693			**
		Metabolism	Glycosyltransferases	K00718			*
			Peptidases and inhibitors	K08676			***
				K07059			*
			Protein phosphatases & associated proteins	K17609			*
		Signaling and cellular processes	Prokaryotic defense system	K19172			*
			Transporters	K21572			*
				K21573			*
				K07221			*
				K16694			***
			Two-component system	K02481			*

b) M06

				KEGG	baseMean	log2FoldChange	sign
BF	Env. Inf. Processing	Membrane transport	ABC transporters	K16784			**
		Signal transduction	Two-component system	K18351			***
				K08475			*
	Metabolism	Carbohydrate metabolism	Pyruvate metabolism	K00156			*
		Metabolism of cofactors and vitamins	Nicotinate and nicotinamide metabolism	K15359			***
		Nucleotide metabolism	Purine metabolism	K01081			*
	NA	Poorly characterized	Function unknown	K09190			***
	Protein families	Genetic information processing	Transcription factors	K07724			**
		Signaling and cellular processes	Secretion system	K12522			***
				K11900			**
				K11896			**
				K11895			*
				K11906			*
			Transporters	K07241			***
NBF	Env. Inf. Processing	Membrane transport	ABC transporters	K17328			*
				K17327			*
	Protein families	Genetic information processing	Transcription machinery	K03090			***
			Transfer RNA biogenesis	K07739			*
		Signaling and cellular processes	Transporters	K16923			**

c) M12

				KEGG	baseMean	log2FoldChange	sign
BF	Env. Inf. Processing	Membrane transport	ABC transporters	K18104			**
				K18892			**
		Signal transduction	Two-component system	K18940			***
	Metabolism	Carbohydrate metabolism	Butanoate metabolism	K03366			**
			Pentose phosphate pathway	K25031			***
		Lipid metabolism	Steroid hormone biosynthesis	K00038			*
	NA	Uncl.: metabolism	Enzymes with EC numbers	K07002			*
				K23978			*
				K13727			***
				K05910			**
	Protein families	Genetic information processing	Transcription factors	K22302			**
		Signaling and cellular processes	Prokaryotic defense system	K19780			*
				K19779			*
			Secretion system	K03195			*
				K03200			*
				K03196			*
				K03194			*
			Transporters	K16323			**
				K08153			***
NBF	Env. Inf. Processing	Membrane transport	ABC transporters	K09692			*
		Signal transduction	Two-component system	K07800			*
	Metabolism	Energy metabolism	Carbon fixation pathways in prokaryotes	K14138			*
	NA	Poorly characterized	General function prediction only	K06878			**
		Uncl.: genetic information processing	Replication and repair	K07467			**
				K07494			*
			Translation	K07576			*
		Uncl.: metabolism	Enzymes with EC numbers	K23753			*
				K04104			*
		Uncl.: signaling and cellular processes	Cell growth	K06412			*
	Protein families	Genetic information processing	DNA repair and recombination proteins	K03660			*
			Transcription machinery	K07315			*
			Transfer RNA biogenesis	K09759			**
		Metabolism	Peptidases and inhibitors	K01267			**
				K01269			**
		Signaling and cellular processes	Transporters	K24950			*
				K10189			*
				K10188			**
				K24948			*
				K10190			**

Suppl. Fig. 20: Hierarchical KEGG functional annotation of genes of highest top30 log fold change rates between breastfed (BF = light green) and non-breastfed (NBF = lavender) infants per time point (months a) M01, b) M06 and c) M12): negative log fold change: red, higher in BF infants; positive log fold change: blue, higher in NBF infants; significant *q*-value indicated by asterisks and base mean indicated by bar charts colored by respective group; abbreviation env. info. processing = environmental information processing.

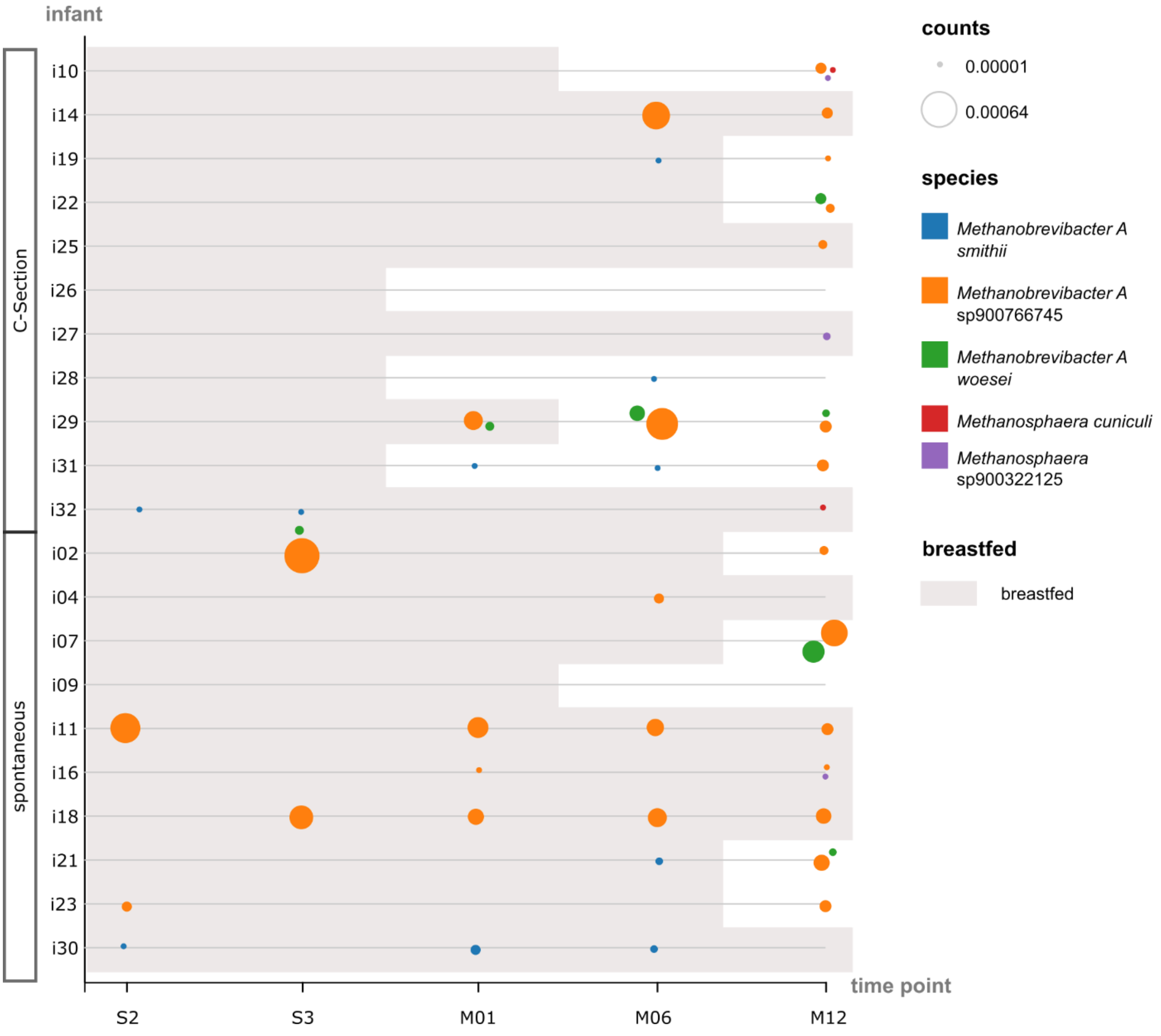

Suppl. Fig. 21: Beeswarm plot on *Methanobrevibacter* species in stool samples for metagenomics sequencing for time points S2, S3 and months M01, M06 and M12; time

points at which the infants were breastfed are underlaid with gray and infants are sorted by their mode of delivery.
